# Imaging the Rewired Metabolism in Lung Cancer in Relation to Immune Therapy

**DOI:** 10.3389/fonc.2021.786089

**Published:** 2022-01-07

**Authors:** Evelien A. J. van Genugten, Jetty A. M. Weijers, Sandra Heskamp, Manfred Kneilling, Michel M. van den Heuvel, Berber Piet, Johan Bussink, Lizza E. L. Hendriks, Erik H. J. G. Aarntzen

**Affiliations:** ^1^ Department of Medical Imaging, Radboud University Medical Centre (Radboudumc), Nijmegen, Netherlands; ^2^ Department of Preclinical Imaging and Radiopharmacy, Werner Siemens Imaging Center, Eberhard Karls University, Tuebingen, Germany; ^3^ Department of Dermatology, Eberhard Karls University, Tuebingen, Germany; ^4^ Department of Respiratory Diseases, Radboudumc, Nijmegen, Netherlands; ^5^ Radiotherapy and OncoImmunology Laboratory, Department of Radiation Oncology, Radboudumc, Netherlands; ^6^ Department of Pulmonary Diseases, GROW – School for Oncology and Developmental Biology, Maastricht University Medical Centre (UMC), Maastricht, Netherlands

**Keywords:** tumor microenvironment, lung cancer, T-cells (or lymphocytes), immunotherapy, metabolism, molecular imaging

## Abstract

Metabolic reprogramming is recognized as one of the hallmarks of cancer. Alterations in the micro-environmental metabolic characteristics are recognized as important tools for cancer cells to interact with the resident and infiltrating T-cells within this tumor microenvironment. Cancer-induced metabolic changes in the micro-environment also affect treatment outcomes. In particular, immune therapy efficacy might be blunted because of somatic mutation-driven metabolic determinants of lung cancer such as acidity and oxygenation status. Based on these observations, new onco-immunological treatment strategies increasingly include drugs that interfere with metabolic pathways that consequently affect the composition of the lung cancer tumor microenvironment (TME). Positron emission tomography (PET) imaging has developed a wide array of tracers targeting metabolic pathways, originally intended to improve cancer detection and staging. Paralleling the developments in understanding metabolic reprogramming in cancer cells, as well as its effects on stromal, immune, and endothelial cells, a wave of studies with additional imaging tracers has been published. These tracers are yet underexploited in the perspective of immune therapy. In this review, we provide an overview of currently available PET tracers for clinical studies and discuss their potential roles in the development of effective immune therapeutic strategies, with a focus on lung cancer. We report on ongoing efforts that include PET/CT to understand the outcomes of interactions between cancer cells and T-cells in the lung cancer microenvironment, and we identify areas of research which are yet unchartered. Thereby, we aim to provide a starting point for molecular imaging driven studies to understand and exploit metabolic features of lung cancer to optimize immune therapy.

## 1 Introduction

Metabolic reprogramming is one of the hallmarks of cancer (1, 2), and the many ways by which cancer cells manipulate their metabolic micro-environment are increasingly being understood. Excellent and comprehensive reviews approaching this topic from the angle of specific cancer types such as non-small cell lung cancer (NSCLC) (3, 4) and head and neck cancer (HNSCC) (5) or from the most common metabolic substrates – oxygen (6), essential nutrients/amino acids (7), lipids/free fatty acids (8), acetate (9) or genetic drivers (10–13) - are available from recent literature.

Alterations in the tumor microenvironmental (TME) metabolites are recognized as important tools for cancer cells to interact with supportive cells in their direct vicinity (14). These supportive cells include endothelial cells, inducing angiogenesis when activated by increased demands for oxygen (15, 16) or cancer-associated fibroblasts driving glycolysis (5, 17). Also, tumor-associated macrophages can modulate glucose metabolism in the TME in favor of cancer progression (18). Interactions between cancer cells and supportive cells are reciprocal in nature (18) and the derivative metabolic phenotypes result from underlying oncogenic mutations (11, 19), pathology (20, 21) as well as from tissue of origin (22). Lung cancer frequently harbors mutations which directly affect cellular glucose metabolism and associated metabolic pathways, as reviewed previously (4). In addition to STK11/LKB1 mutations (23, 24), mutations in the PI3K (phosphoinositide-3-kinase)-AKT-mTOR (mammalian target of rapamycin) pathway (23), the oncogenes RAS, c-MYC, and master regulator HIF-1α (hypoxia inducible factor-1α), or the tumor suppressor gene TP53 are known to reprogram lung cancer metabolism.

**Figure 1:**
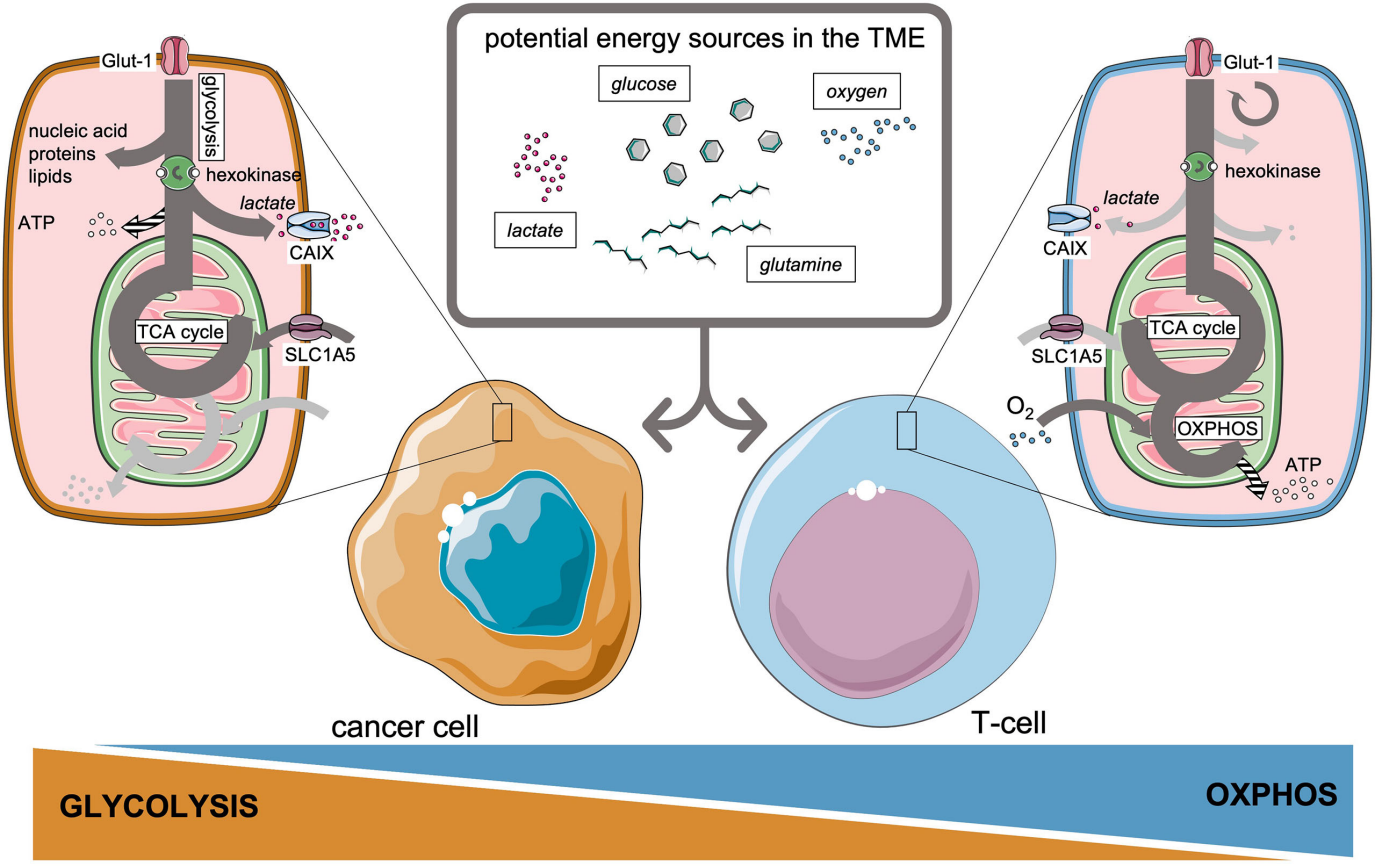
Preferential metabolic pathways and potential energy sources available in the tumor microenvironment for cancer cells and T-cells.

By modulating metabolic pathways and depriving the TME from essential nutrients, cancer cells create unfavorable conditions for invading adaptive immune cells (20, 25–27). To execute their effector functions, T-cells should undergo rapid metabolic reprogramming (28, 29), which mainly involves upregulation of aerobic glycolysis by CD28 co-stimulation, acting through PI3K and Akt pathways (30, 31), very much alike the Warburg effect in cancer cells (32). Yet on the longer term, a sustainable memory T-cell response requires a distinct metabolic profile that relies on oxidative phosphorylation and intact mitochondrial function to prevent T-cell exhaustion (33–35). **Figure 1** introduces the main potential sources of energy available in the TME, which will be discussed in this review, and the preference of cancer cells and T-cells to perform glycolysis or oxidative phosphorylation, respectively. Glucose metabolism therefore illustrates that nutrient availability represents a highly conserved fundamental framework to guide decisions on cell survival or apoptosis (36), a process which is continuously taking place in the TME. Next to glucose metabolism, other basal metabolic pathways involving amino acids (7) like glutamine (37) and lipids (8, 38, 39) are reported to affect T-cell immunity.

Cancer-induced metabolic changes in the TME not only favor cancer progression and immune suppression but can also be a limiting factor concerning treatment efficacy. The most studied example in lung cancer is the adverse role of lack of oxygen availability blunting radiotherapy efficacy (40–42). Similarly, blocking adaptive metabolic pathways renders standard chemotherapy more effective in lung cancer (43, 44). Also, development of resistance to targeted therapies is related to plasticity in metabolic pathways associated with Kirsten rat sarcoma viral oncogene homolog (KRAS) mutations in NSCLC (45, 46), a vulnerability which can be exploited in combination treatments (47). More recently, the metabolic determinants of immune checkpoint inhibition are being understood (48). For example, glucose consumption by cancer cells might be a metabolic adaptation to restrict T-cell effector function (26, 49). Furthermore, blocking programmed death ligand-1 (PD-L1) on cancer cells reduces their glycolysis rate by inhibition of mTOR-related pathways, which would permit T-cells to exploit their glycolytic capacity and restore IFN-γ production (26). Acknowledging the intertwined roles of immune checkpoint molecules, both on cancer cells and T-cells, in immune signaling and regulation of cellular metabolism, this is now an active area of research (50, 51). Ongoing onco-immunology studies on checkpoint inhibitors search to utilize the effect of checkpoint molecule inhibition on cancer cell metabolism, as adjunct to enhancing immune cell function (52, 53).

In addition, onco-immunological treatment strategies emerge that employ the metabolic vulnerabilities of cancer cells, especially at the level of mitochondria (54–58). These strategies include enzymatic drugs that interfere with dominant metabolic pathways in the TME (59), such as metformin, atovaquone, glucose (60), indoleamine 2,3-dioxygenase (IDO inhibitors), glutamine inhibitors (37) and AKT-mTOR inhibitors (27). The efficacy of mitochondrial targeting drugs indicates that oxidative phosphorylation remains important for adenosine-triphosphate (ATP) production in a multitude of tumors, including NSCLC (61, 62).

Tumor senescence represents another important tumor suppressor mechanism (63), apart from apoptosis, embanking cancer cell proliferation as well as malignant progression. Tumor senescence implies stable cell-cycle arrest induced by cellular stress associated with alterations in gene expression patterns, a metabolic shift towards a more glycolytic state and a proinflammatory secretory phenotype (64, 65). Multiple anticancer therapies such as chemotherapy, radiotherapy and cancer immunotherapies are applicable to induce irreversible tumor senescence. Thus, tumor senescence has to be taken into account as an essential component in the treatment of cancer.

**Figure 2:**
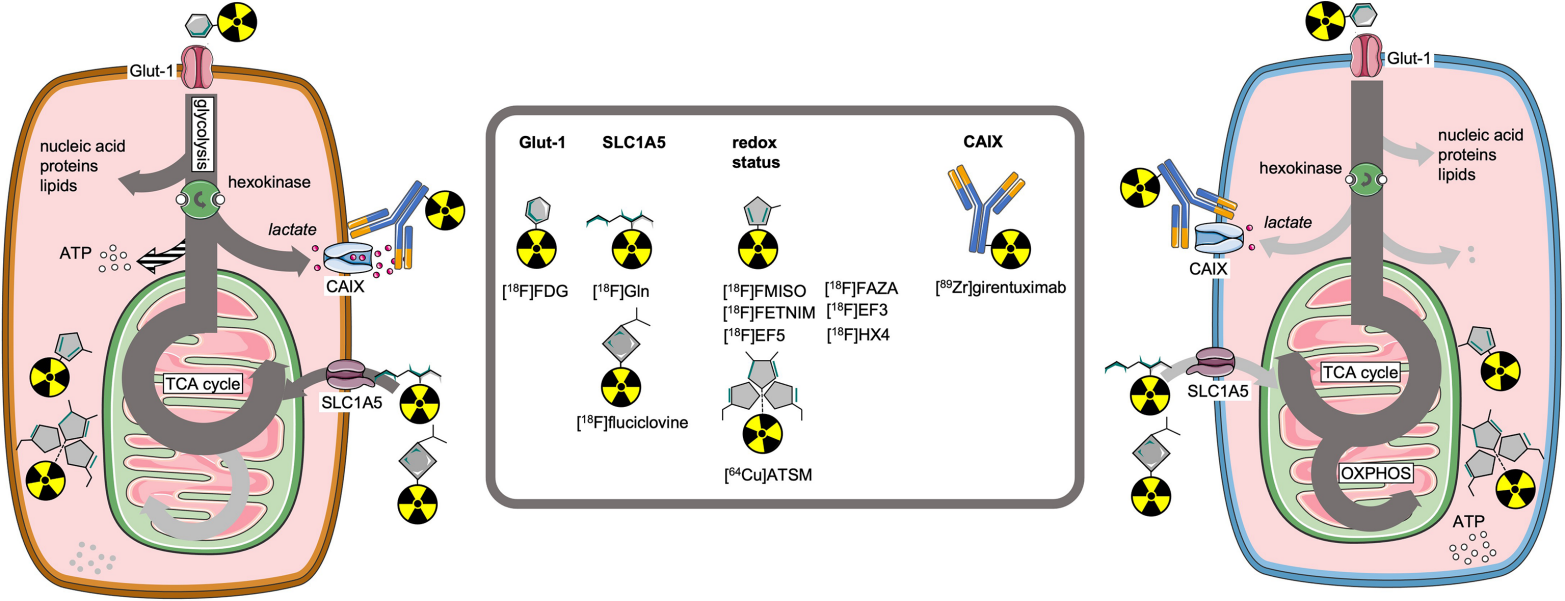
Molecular imaging tracers to visualize key receptors and pathways involved in cell metabolism.

PET imaging has developed a wide array of tracers targeting metabolic pathways, originally intended to improve cancer detection and staging (66, 67). Paralleling the developments in understanding metabolic reprogramming in cancer cells, as well as its effects on T-cells, a wave of additional imaging tracers has been published (68, 69) (**Figure 2**).

Definitively, imaging can contribute to more effective anti-cancer therapies (70–72), as it assesses functional processes with high sensitivity and, if applied longitudinally, can monitor treatment effects on an individual patient basis. This adds important insights to immunohistochemistry that can provide a detailed but static insight in the expression of transporters, enzymes and other molecular markers involved in metabolic pathways. Although the methodology allows quantitative assessment of these functional processes this is limited to accessible lesions and hampered by sampling errors. In addition, molecular imaging facilitates evaluation of intra-tumoral regional differences, a critical aspect for the net treatment efficacy (73) which cannot be assessed with invasive sampling procedures such as biopsies. As metabolic adaptation of T-cells can have tissue-specific determinants (74) which might differ from *in vitro* experiments (75), the wide field of view of PET imaging is a critical asset in this domain of research.

However, molecular imaging tools that probe metabolic processes are yet under-utilized in the perspective of immune therapy development. In this review, we provide an overview of currently available PET imaging tools for clinical studies and discuss their potential roles in the development of effective immune therapeutic strategies in lung cancer. We report on ongoing efforts that include PET/CT to understand the outcomes of interactions between lung cancer and T-cells in the tumor microenvironment, and we identify areas of research which are yet unchartered. Thereby, we aim to provide a starting point for molecular imaging driven studies to understand and exploit metabolic features of tumor environment to optimize immune therapy.

## 2 Glucose Metabolism

### 2.1 Glucose Metabolism in Cancer Cells

The most studied metabolic phenomenon in cancer is its tendency to increase its’ rate of glycolysis in adjunct to oxidative phosphorylation, despite the presence of sufficient levels of oxygen in the TME. This feature of cancer metabolism is called the Warburg effect, named after the German scientist who first described this (76). Although glycolysis is less efficient in producing ATP, it does generate increased levels of additional metabolites for the biosynthesis of ribose, glycosylation precursors, amino acids, and lipids (77, 78). Inefficient ATP production would only be problematic in scarcity of nutrients, which is in general not the case in cancer. Therefore ‘aerobic glycolysis’ means a survival advantage for cancer cells in terms of increased anabolism and avoidance of oxidizing precious carbon-carbon bonds (79).

To meet their greatly enhanced demand for glucose under conditions of ‘aerobic glycolysis’, cancer cells have upregulated levels of the key glucose transporter 1 (Glut-1) on cell membranes (10) and associated hexokinases. Hexokinases are enzymes that phosphorylate six-carbon sugars, primarily glucose, by transferring a phosphate group from ATP to its’ substrate. As phosphorylation charges the hexoses, it is trapped intracellularly and available for further metabolic processes, resulting in a stable down-slope gradient that drives glucose transport into the cell. For this reason, hexokinase activity is the rate limiting step for most metabolic pathways involving glucose. The isoform hexokinase II (or hexokinase B) is the dominant isoform in many cell types (80), including most cancers, and located at the outer mitochondrial membrane to have direct access to ATP (81). Upregulation of aerobic glycolysis results in an increase of pyruvate, which is further metabolized into lactate (77). Intracellular lactate is transported out of the cell, along with protons, *via* monocarboxylate transporters (e.g., MCT-1 and MCT-4) into the TME. In addition to lactate shuttles, intracellular acidification is prevented by carbonic anhydrase 9 (CAIX), a transmembrane metalloenzyme that facilitates secretion of acids produced under oxidative stress. Indeed, it has long been noticed that tumors often have an acidic environment (82).

### 2.2 Regulation of Metabolic Reprogramming in Lung Cancer

Metabolic reprogramming in cancer is partly due to oncogenic activation of signal transduction pathways and transcription factors, HIF-1α is a master regulator of glycolysis and the pentose phosphate pathway (20, 83, 84). In lung cancer, oncogenes and pathways divert intracellular glucose flux towards increased usage of glucose into the hexosamine biosynthesis, required for protein glycosylation and pentose phosphate pathway [reviewed in (4)]. Well-studied signaling pathways, including PI3K/Akt/mTOR and RAS/RAF/MEK/MAPK, with high prevalence in lung cancer, associate with increased glycolysis as well as metabolic plasticity, by initiating compensatory mechanisms and facilitating alternative metabolic sources, e.g., amino acids, nucleotides or fatty acid biosynthesis and macropinocytosis. At a transcriptional level, the transcription factor nuclear factor erythoid-2-related factor (NFE2L2/Nrf2) is identified as one of the main regulators of metabolic reprogramming in lung cancer, and its activity is associated with poor survival (85).

Epigenetic mechanisms also contribute to the regulation of gene expression involved in cancer metabolism (86). Disruption of the epigenome is present in cancer cells, including DNA methylation, histone proteins and histone modification enzymes, as well as proteins that regulate the function of metabolic enzymes (87). Reciprocally, activity of histone and DNA modifying enzymes regulates the expression of metabolism-associated genes, leading to a complex interplay between metabolism and epigenetic during cancer progression (88). Understanding the relation between metabolism, signaling pathways and epigenetics may open new avenues for anti-cancer immune therapy (89), which will be discussed later.

### 2.3 How Glucose Consumption by Cancer Cells Affects T-Cells

Upon activation, naïve T-cells also undergo metabolic adaptation to meet the increased bioenergetic demands associated with proliferation and effector function (29, 90–93). In contrast to static cancer cells, which can thus invest in creating a favorable metabolic niche, effector T-cells migrate through the body and are merely passengers who need to adapt to changing environmental conditions, from well-supplied lymph nodes and spleen to rather oxygen and nutrient deprived cancer lesions (94). In general, nutrient competition between cells strongly influences cell fate (36, 95) and function (90). More recently, this interplay between cancer cells and immune cells has been reviewed (51, 96, 97). Aerobic glycolysis is not required for activation or proliferation during early stages of T-cell activation (98), however, it is essential for optimal T-cell effector function in the TME (99, 100). *In vitro* models previously demonstrated that cancer cells outcompete T-cells for glucose, directly restricting cytokine mediated anti-cancer immunity (101). Also *in vivo*, tumor infiltrating CD8+ T-cells face restricted glucose availability, which consequently hampers increased rate of glycolysis by restricted mTOR activity and thus reduced IFN-γ production (98, 102).

In addition to direct competition for glucose, limiting the magnitude of aerobic glycolysis in T-cells, high lactate excretion by cancer cells further suppresses T-cell effector functions (103–105), directly correlated it to reduced survival rates in e.g., head and neck cancer (106). The acidic TME inhibits both T-cell trafficking and cytotoxicity (102, 103, 107) and sheds new light on the role of lactate as immune metabolic mediator (14). The enzyme lactate dehydrogenase A (LDHA) which converts pyruvate into lactate, not only plays a central role in cancer cell aerobic glycolytic capacity but exerts similar function in T-cell function through PI3K signaling (31, 108).

The costimulatory molecule CD28 on T-cells, ligating to CD80 during antigen-specific activation, induces this PI3K signaling (30), resulting in increased expression of Glut-1. By facilitating glycolysis increase, CD28 signaling prepares T-cells to anticipate on changing metabolic demands associated with sustained effector functions. This necessary metabolic switch is furthermore under the control of inhibitory members of the CD28 superfamily (mainly PD-1 expression), with the intend to delicately control T-cell activation (97, 109–111). PD-1 on T-cells is mostly studied as an exhaustion marker, induced by chronic antigen exposure and endurable stages of activation. Its increased expression on T-cells indicates a critical stage of T-cell development, at the verge of going in retraction and clearance (109, 112). The expression of PD-L1, by cancer cells and myeloid derived suppressor cells in the TME not only suppresses cytotoxic effector function of T-cells, but it also entangles the metabolic reprogramming of T-cells *via* ligation of PD-1. PD-1 ligation suppresses the ability of T-cells to perform glycolysis and glutaminolysis, thus pushing T-cells further towards retraction. Therefore, one of the effects of therapeutic monoclonal antibodies targeting CTLA-4 (interacting with CD28) and PD-L1 (interacting with PD-1), is allowing T-cells to maintain their increased glycolytic and glutaminolytic capacity to execute anti-cancer effector functions in the TME (113, 114).

### 2.4 Imaging Targets Related to Glucose Metabolism

The most widely applied tracer to image the upregulation of glycolysis is 2’-deoxy-2’-[^18^F]fluoro-D-glucose ([^18^F]FDG). [^18^F]FDG is extensively used for the detection of primary tumors, metastases and recurrences, and monitoring responses to anti-cancer treatments (115–117). [^18^F]FDG uptake by glycolytic cancer cells is directly related to upregulated levels Glut-1 transporters (118) and hexokinases activity (119). Consequently, levels of [^18^F]FDG uptake also correlate with increased levels of derivates of the glycolytic pathway; pyruvate and lactate (120).

#### 2.4.1 [^18^F]FDG to Characterize the Tumor Immune Microenvironment

**Figure 3:**
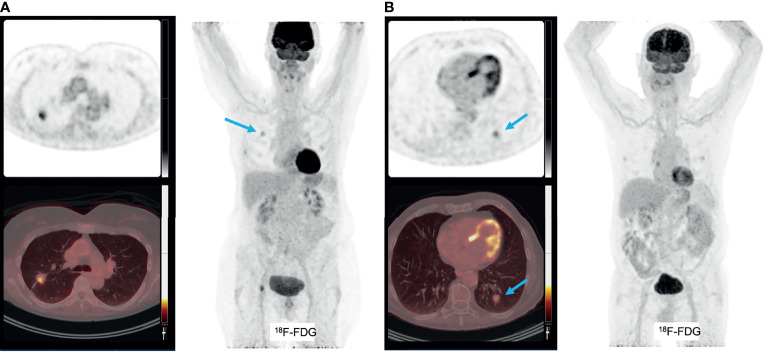
**(A)** patient with pT2bN0 well-differentiated primary adenocarcinoma of the right upper lobe, with markedly increased [^18^F]FDG uptake **(A)**. This tumor was PD-L1 negative. Molecular analyses: mutations found in KRAS (p.G13C), BRAF (p.G464V) and STK11, no amplifications, no micro-satelite instability. An additional example of a patient with pT1bN0 well-differentiated primary adenocarcinoma of the left lower lobe, with faint [^18^F]FDG uptake **(B)**. This tumor was PD-L1 negative and molecular analyses detected mutations in KRAS (p.G12A), no amplifications and no micro-satellite instability.

Given the reciprocal relation of glucose metabolism between cancer cells and T-cells, several studies investigated the relation between [^18^F]FDG-uptake, as parameter for glycolysis in the TME, and expression levels of immune checkpoint molecules and presence of CD8+ T-cells. Independent of the well-known higher [^18^F]FDG-uptake in squamous cell histological subtypes as compared to adenocarcinoma in NSCLC (20), some studies found a trend towards higher SUV_max_ and SUV_mean_ in lung cancers with increasing numbers of CD8+ T-cell numbers and increased expression of PD-1 (121). Not surprisingly, CD8+ T-cells and PD-1 expression were highly intercorrelated and overlapping their positive correlation with [^18^F]FDG-uptake. However, there was no such relation between [^18^F]FDG-uptake and presence of tumor-associated macrophages, measured by CD68 staining, or PD-L1 expression (**Figure 3**). Others did find a positive relation between [^18^F]FDG uptake and PD-L1 expression on immunohistochemistry in patients with NSCLC (122–124). High maximum [^18^F]FDG uptake in NSCLC seemed prognostic for poor disease free survival (121), but it might be predictive for a favorable response to immune checkpoint inhibition (125).

In contrast, high levels of [^18^F]FDG uptake by cancers cells, corresponding with upregulated expression of glycolysis-related genes, was correlated with reduced numbers of CD8+ T-cells, increased T-cell exhaustion gene signatures and higher levels of PD-L1 in NSCLC by others (126), which potentially can stratify patients for subsequent immune checkpoint inhibition. This negative trend has also been observed in HNSCC using a systems biology approach, correlating omics data with histopathological data; CD8+ T-cell numbers were inversely correlated with HIF-1α and EGFR regulated aerobic glycolysis (127). This was confirmed by a similar approach in HNSCC demonstrating reduced numbers and activation status of CD8+ T-cells as well as myeloid cells with increasing [^18^F]FDG uptake (128), and renal cell carcinoma (129, 130).

#### 2.4.2 [^18^F]FDG to Monitor Response to Immune Checkpoint Inhibition

Decrease in [^18^F]FDG uptake in melanoma, renal cell or lymphoma lesions within 3 months after start of immune checkpoint inhibition was correlated with a favorable clinical outcome at 1 year (131). Several additional studies confirmed the role of [^18^F]FDG to monitor response to immune checkpoint inhibition in patients with advanced melanoma treated with CTLA-4 inhibitors (132–134) and advanced NSCLC patients under PD-1 inhibition (124) These studies suggest that [^18^F]FDG may serve as a predictor of response of immune checkpoint inhibition (132, 133), as long as immune therapy related response patterns are taken into account, e.g. appearance of new lesions or initial limited increase in tumor burden not per se define progression (135, 136). At this moment several clinical trials are ongoing with [^18^F]FDG as a biomarker of therapeutic responses to immunotherapy in various cancer types, including thoracic cancer (NCT02608528), NSCLC (NCT02753569 and NCT04082988), and melanoma (NCT04272658). These current studies, generally hinting at a conventional role for [^18^F]FDG PET/CT to assess cancer responses to immune therapy, presumably demonstrates that in the TME cancer cell glycolysis largely outcompetes glycolysis by tumor-infiltrating immune cells.

However, early signs of increased T-cell activity, by upregulated [^18^F]FDG-uptake as a surrogate for increased glycolysis, at sites distant from the TME are readily visualized using [^18^F]FDG PET imaging (137). Immune related adverse events like thyroiditis are associated with favorable clinical outcome (131). In addition, systemic immune activation, linked to increased glycolysis in hematopoietic bone marrow and secondary lymphoid organs such as the spleen, show a positive correlation with favorable response to immune checkpoint inhibition (138–141).

## 3 Oxygen Availability

### 3.1 How Oxygen Availability Affects Cancer Cells

Glycolysis, even when increased, contributes relatively little to cellular ATP content; its majority is provided by oxidative phosphorylation in mitochondria, which requires oxygen in the electron transport chain (142). Although oxygen availability is only limiting mitochondrial electron transport chain at very low levels (<0.07% oxygen) (143, 144), its lowering levels are sensed carefully. Through HIF1a activation, hypoxia promotes glycolysis in addition to increasing oxidative phosphorylation (145, 146). Imbalances in oxygen levels occur in a range of physiological conditions, e.g., wound healing (147), and disease conditions, e.g. chronic obstructive pulmonary disorders (148). In cancer however, the chaotic tissue vascularization results in *chronic* diffusion-limited hypoxia as well as *acute* perfusion limited hypoxia (15). Enduring long-term hypoxia results in additional oxidative stress caused by increased levels of reactive oxygen species (ROS) produced from mitochondrial complex III in cancer cells (149, 150) Excessive levels of intracellular ROS can cause oxidative damage to intracellular lipids, protein and DNA, which might reciprocally drive diversification of cancer phenotypes (151) but at a given point culminates in cell cycle arrest and apoptosis (152).

### 3.2 Lung Cancers’ Response to Hypoxia

Intracellular oxygen homeostasis is regulated by the hypoxia-inducible factor (HIF), a heterodimer that is composed of two subunits HIF-1α and HIF-1β (153). HIF-1α transcriptional activation is triggered by short-term hypoxia of 2–24 h with oxygen tensions <0.1% oxygen, while the isoform HIF-2α activation occurs under milder hypoxic conditions (<5% oxygen). Under *normoxic* conditions, HIF-1α is degraded under control of the von Hippel-Lindau (VHL) protein. Under *hypoxic* conditions, HIF-1α is stabilized and binds to HIF-1β before translocating to the nucleus to bind the hypoxia response elements (HRE) that targets genes involved in intracellular acid-base balances, such as carbonic anhydrase IX (CAIX) (154). Furthermore, it induces transcription of genes involved in glycolysis (including Glut-1, hexokinases (155, 156)), angiogenesis and proliferation (157). While HIF initiates increase in glycolysis, the glycolytic products pyruvate and lactate in their turn induce HIF-1α accumulation, indicative of a sustained feed-forward mechanism driving tumor metabolism towards glycolysis (158, 159).

Another result of HIF-1α upregulation is increased expression of CAIX as is described above (160) and MCT-4 (161). The interaction of CAIX with MCT-1 and MCT-4 is linked to acidification of the TME (162) and associated with poorer prognosis (163) and immune suppression (164). However, whether CAIX expression can serve as a surrogate for tumor hypoxia is debatable (165) and clinical studies on CAIX expression in lung cancer are scarce.

As VEGF is the main mediator of angiogenesis in many types of cancer to cater to chronic hypoxic conditions and as VEGF is under control of HIF signaling, it is also aberrantly expressed in lung cancer (166), in particular in adenocarcinoma (167). The level of VEGF expression is correlated with micro-vessel density and development of hypoxia and is involved in the so-called secondary vascular growth phase (168–170). It is suggested that, although VEGF expression stimulates angiogenesis, the disorganized and immature features of newly formed blood vessels in fact sustain the presence of intra-tumoral regions of hypoxia (171). Consequently, VEGF expression is in most studies correlated to a worse survival in NSCLC (172).

In parallel to oxidative stress, oncogenic mutations in lung cancer can also induce HIF activation, e.g., phosphatase and tensin homolog (PTEN), PI3K/Akt/mTOR pathway (152, 173), or epigenetic alterations (174, 175). As a consequence of increased HIF-1α signaling, PD-L1 expression on lung cancer cells increases (176–179).

### 3.3 How Oxygen Availability Affects T-Cells

On a general note, cancer cells show a greater metabolic plasticity than effector T-cells and have evolved to manipulate the host TME to their benefit, which enables them to utilize a variety of alternative metabolic pathways and substrates also under hypoxic conditions. Consequently, these alternative metabolic pathways often come with side-products, such as ROS, which require an additional set of processes to compensate for collateral damage. Although T-cells have differential metabolic preferences throughout their lifespan, they display limited plasticity or compensating pathways to deal with the ‘metabolic waste’ from cancer cells, resulting in ‘exhausted’ states in the TME (93, 101). For example, high levels of ROS in the TME are toxic for T-cells (180–182). Central to these effector function insufficiencies is mitochondrial function, which shows hyperpolarization, fragmentation, and increased ROS production in the TME (35, 59, 114, 130, 183). Furthermore, intratumoral hypoxia also limits T-cell migration away from the blood vessels into the tumor micro-environment, creating hypoxic immune privileged niches within the tumor (184). Thus, in addition to the limited availability of glucose itself, hypoxia further restricts T-cells’ capacity to perform aerobic glycolysis (paragraph 2.3), hampers T-cell infiltration and hypoxia-related waste products directly affects T-cell viability.

### 3.4 How T-Cells Respond to Hypoxic Conditions

As mentioned above, induction of glycolysis is essential for T-cell effector functions and this induction is under control of mitochondrial ROS signaling and HIF-1α under *normoxic* conditions (6, 94) In particular Th17, Th1 CD4+ T-cells and CD8+ T-cells rely on increased glycolysis, whereas regulatory T-cells show less glycolysis dependency (93). For example, upon activation of CD3/CD28 on CD8+ T-cells, the expression of HIF-1α increases *via* PI3K/AKT/mTOR pathways to allow for increased glycolysis and effector functions such as IFN-γ and TNF-α secretion (185, 186). Under *hypoxic* conditions, HIF-1α induces downregulation of IFN-γ production by Th1 cells (187). These observations suggest a complex dual role for HIF-1α signaling in T-cells, which is environment and stimulus dependent. While glycolysis is required for T-cell effector functions, glutaminolysis and the pentose phosphatase pathway are necessary for biosynthesis. T-cell receptor triggering increases amino-acid transporters, along with upregulation of glucose metabolism, and therefore contributes to T-cell activation. However, given its different role glutaminolysis does not compensate for the dependency on glycolysis under hypoxic conditions. In fact, depletion of amino acids in the TME such as L-arginine by myeloid derived suppressor cells, inhibits T-cell proliferation (101). Perhaps the most important alternative metabolic pathways for T-cells in the TME to meet their metabolic demands is fatty acid oxidation (188). T-cell effector function is partially preserved by upregulating PPAR-a signaling to metabolize fatty acids under hypoxic and hypoglycemic conditions (39). Promotion of fatty acid metabolism could synergize with PD-1 blockade to control tumor growth, as shown in a preclinical melanoma model.

### 3.5 Hypoxia Blunts Efficacy of Anti-Cancer Treatment

Several preclinical and human studies have identified roles of hypoxia in blunting treatment efficacy, as a longstanding notion across cancer types (106, 189, 190), and in particular in radiotherapy (191). Radiosensitivity starts to decrease at oxygen tensions below 2% oxygen, most directly by decreased availability of molecules for radiolysis to produce ROS by ionizing radiation. The hypoxia found in cancer also leads to downregulation of the type I IFN pathway, while this pathway is necessary for an adequate immune response.

In addition to directly reducing the therapeutic potential of ionizing radiation, the downregulated type I IFN pathway due to hypoxia impairs immune activation upon immunogenic cell death, a phenomenon that is observed for radio- as wells as chemotherapy (192). Furthermore, regulatory T-cells and memory CD8+ T-cells largely depend on oxidative phosphorylation, which is also restricted under hypoxia (101), and at least partly explains the arduous task of immune activation in hypoxic tumor regions. Lastly, the disturbed vascularization in tumors is known to hamper the intra-tumoral delivery of therapeutic agents, resulting in sub-therapeutic intra-tumoral concentrations (193, 194). To this end, anti-angiogenic treatments have been introduced in adjunct to radiotherapy (191, 195) targeted- or chemotherapy (196, 197). The overall results over combination treatments targeting VEGF in NSCLC so far have been disappointing (198).

More recently, other processes involving tumor vasculature associated endothelial cells have been identified, which act in addition to the typical vessel sprouting induced by hypoxia-driven or mutation-driven PI3K/Akt signaling. These processes include vessel co-option and vascular mimicry and may partly explain previous ambiguous results of combination treatments in NSCLC. These alternative angiogenic process also illustrate the complex network between NSCLC, supporting stromal cells, such as endothelial cells and pericytes, and mobile immune cell populations. It is generally accepted that angiogenesis factors drive an immune suppressive microenvironment (16, 184). In preclinical models VEGF inhibition resulted in enhanced T-cell infiltration and improved anti-cancer immune responses (199) and help the induction of tertiary lymphoid structures. These studies sparked the interest in combining anti-angiogenic treatment with immune checkpoint inhibitors (200).

### 3.6 Imaging Targets Related to Oxygen Availability

**Figure 4:**
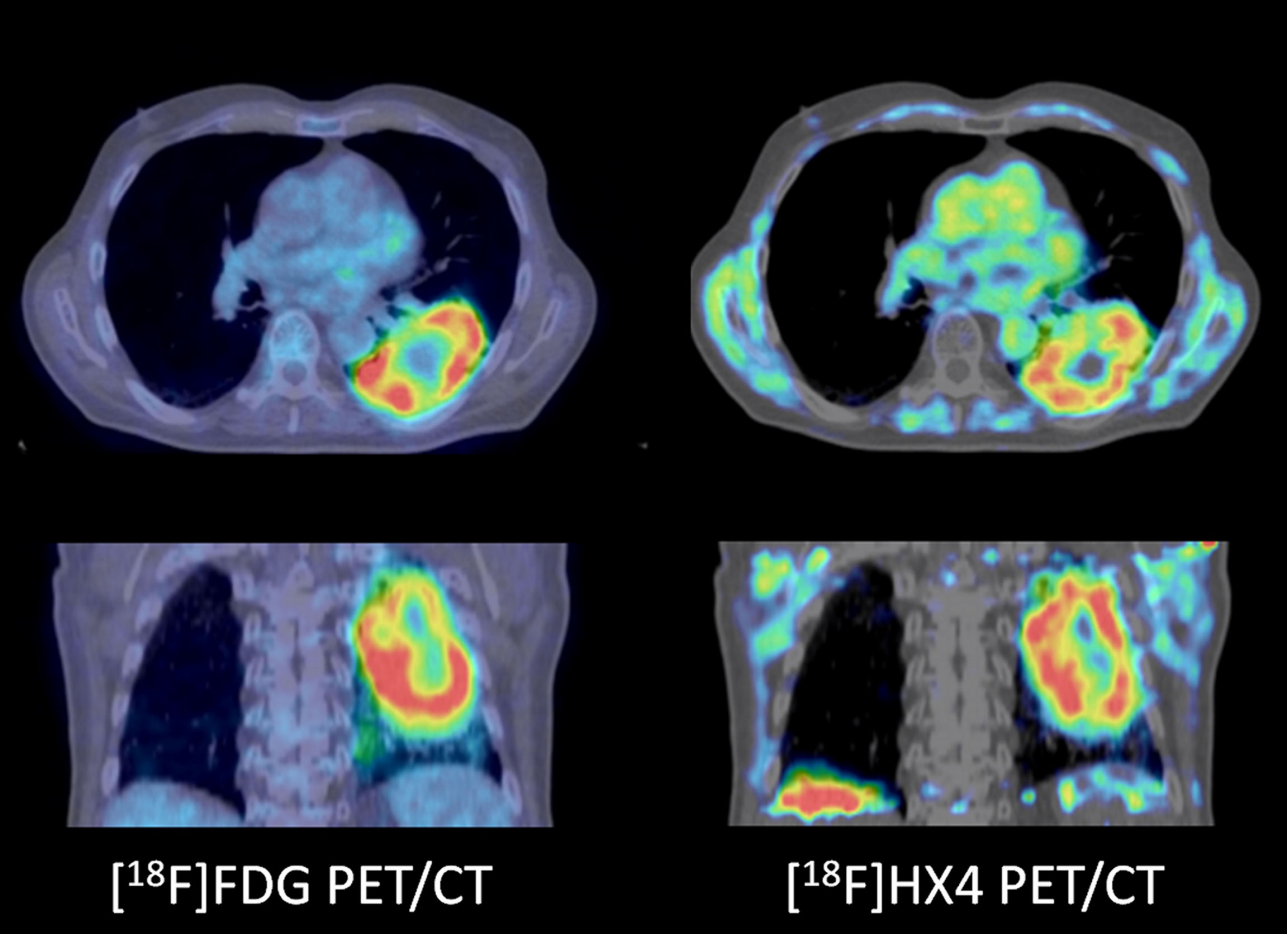
A patient with a cT3N2M0 non-small cell lung cancer not otherwise specified. PD-L1 status or molecular analyses was not performed. The tumor showed increased [^18^F]FDG uptake (left panels) as well as increased [^18^F]HX4 uptake (right panels), indicative of increased hypoxic stress. Note the regional differences of metabolic profiles in the tumor, for example the cranial part ([^18^F]HX4 more than [^18^F]FDG) versus the caudal part (both [^18^F]HX4 and [^18^F]FDG increased).

Most of the current clinical hypoxia PET tracers are ^18^F-fluorinated nitroimidazole compounds, which target the altered redox status in cancer cells and its uptake is increased in hypoxic cells. The mechanism of fluorinated nitroimidazoles is based on an oxygen-reversible single-electron reduction of the nitro group, resulting in the formation of oxygen radicals which covalently bind to macromolecules in hypoxic cells (201), resulting in intracellular trapping of the tracer. In clinical setting, ^18^F-fluoromisonidazole ([^18^F]FMISO) is the most widely used tracer for hypoxia (202, 203). However, [^18^F]FMISO has slow clearance and low tumor uptake (204), which led to the development of second generation 2-nitroimidazole tracers, [^18^F]fluoroazomycinarabinofuranoside ([^18^F]FAZA), [^18^F]FETNIM, [^18^F]EF3, [^18^F]EF5 (205). Even a third generation 2-nitroimidazole hypoxia tracer ([^18^F]HX4, **Figure 4**) has been developed and clinically tested, showing more favourable pharmacokinetic and clearance properties than other ^18^F-fluorinated nitroimidazole compounds (206, 207). [^18^F]HX4 is for these reasons favored over previous hypoxia tracers for response monitoring to (chemo-)radiation therapy (208–210).

Besides nitroimidazole analogues, other compounds that target the redox status in cancer cells are diacetyl-bis(N (4)-methylthiosemicarbazone (ATSM), radiolabeled with different copper radioisotopes, or ionic copper (II) (211). [^64^Cu]ATSM has several advantages over other nitroimidazole derivative hypoxia markers, including rapid tumor uptake and faster clearance from normoxic tissues (212). Several studies in lung cancer have shown that radiolabeled ATSM targets different regions within a tumor as compared to [^18^F]FDG (213, 214), and enable prediction of response to radiotherapy (215). Similar findings have been observed in patients with locally advanced HNSCC, in which [^62^Cu]ATSM was evaluated as a predictor of response (216), with results paralleling [^18^F]HX4.

As hypoxia upregulates expression of CAIX on cancer cells, multiple radiotracers have been identified and tested pre-clinically for the imaging of CAIX, such as the anti-CAIX monoclonal antibody (mAb) G250, girentuximab (cG250), girentuximab antibody fragment Fab’ and F(ab’)2, and more recently affibody molecules. In a recent comparative preclinical study, the affibody ZCAIX:2, antibody fragment girentuximab-F(ab’)2, and a complete antibody-based tracer were evaluated for imaging upregulation of CAIX in head and neck cancer xenograft models (217). Radiolabeled girentuximab, girentuximab Fab’ and F(ab’)2 fragments are also evaluated in human colorectal cancer xenografts (218). According to these studies the complete girentuximab IgG tracer showed the most promising results in both human tumor xenografts. In the clinical setting, the chimeric mAb girentuximab is mostly tested for targeting of CAIX in clear cell renal cell carcinoma (ccRCC) (219), but no clinical studies have been performed on primary lung cancer.

Alternatively to molecular imaging tracers with a particular target in a hypoxia related pathway, multi-modal imaging that combines tissue characteristics, using dynamic contrast enhanced CT, and glucose metabolism, using routine [^18^F]FDG PET, was shown to accurately predict the presence of intra-tumoral regions with hypoxia (as defined by [^18^F]HX4 accumulation (208)).

### 3.7 Hypoxia Imaging to Monitor Response to Immune Checkpoint Inhibition

As the agreement among different hypoxia-related tracers for PET imaging, or agreement with regional [^18^F]FDG uptake in NSCLC is modest (220–224), it remains critical to obtain tissue validation or solid clinical endpoints (225) when incorporating hypoxia tracers in NSCLC studies. However, the most extensively tested hypoxia-related imaging tracer for response prediction in the clinical setting is [^18^F]FMISO. In early stage NSCLC, the combined pattern of high [^18^F]FDG and high [^18^F]FMISO uptake was associated with an increased risk of recurrence after stereotactic radiotherapy (226). Such metabolic profile based in molecular imaging could help in guiding intensity-modulated treatment, as demonstrated in locally advanced NSCLC to avoid deleterious effects on organs-at-risk (227–229).

No studies have yet been performed regarding hypoxia imaging and immunotherapy in NSCLC, but in HNSCC [^18^F]FMISO imaging was used pre-clinically in combined anti-PD-1 and anti-CTLA-4 treatment to monitor changes in the TME during treatment. Preliminary data shows the potential to predict response to checkpoint blockade with anti-PD-1 and anti-CTLA-4 therapy [Reeves et al. *J Nucl Med* 2020, volume 61, supplement 1; 407, meeting report]. In HNSCC patients, increased lymphocyte infiltration is seemingly determined by a hypoxia-dependent response to chemoradiation (230), and persistent hypoxia during definitive chemoradiation treatment correlated with persistent PD-L1 expression and reduced outcomes (230), illustrating the potential of hypoxia related imaging to probe the tumor microenvironment. Several clinical trials are ongoing with [^18^F]FMISO as read-out in radiotherapy trials. One phase IB/II trial is ongoing to examine the feasibility and safety of the combination of two immune checkpoint inhibitor therapies (nivolumab and ipilimumab) in the neoadjuvant setting in resectable HNSCC. In this study, hypoxia measured by [^18^F]FMISO PET imaging is investigated as determinant for the effect of immune checkpoint inhibitors on the intratumoral T cell capacity (NCT03003637).

## 4 Glutamine Metabolism

### 4.1 Glutaminolysis in Cancer Cells

In addition to glucose, most tumor types also display increased uptake of amino acids, such as glutamine, to meet their high demands in biosynthesis and macromolecular synthesis (79, 231). Glutaminolysis is the intracellular conversion of glutamine to glutamate by glutaminase (GLS). This process is facilitated by the upregulation of the alanine-serine-cysteine transporter 2 (ASCT2, also known as SLC1A5) receptors in different cancer types (231), including lung cancer (232). In particular under low-oxygen conditions, glutamine becomes a carbon source for proliferating cancer cells to perform lipogenesis *via* reductive carboxylation (142), taking over up to 80% of *de novo* lipogenesis in A549 lung carcinoma cells (233). *Via* several other routes, glutaminolysis provides a back-up for metabolic pathways that are usually sustained by glucose metabolism; by providing a source of NADPH (234) and the glycolytic intermediate PEP when gluconeogenesis can no longer be performed (235). Thus, increased glutaminolysis in most cancer types illustrates their metabolic plasticity and provides an alternative source to glycolysis for intracellular bioenergetics. This dual reliance of lung cancer is further illustrated by the upregulation of glutaminolysis once glycolysis is suppressed (236).

Another important role for glutamate in cancer cells is its conversion into glutathione, a critical intracellular redox buffer, which is necessary to counteract the oxidative stress inflicted by aerobic glycolysis (237).

Similar to glycolysis, increased glutaminolysis is driven by increased signaling in the PI3K and/or Akt, which results in increased signaling of mTOR. Lung cancer frequently harbors mutations in the receptor tyrosine kinases or further downstream (238), and some of the metabolic heterogeneity observed in lung cancer cell lines can be attributed to mutations in KRAS or Trp53, apart from their histological subtype being adenocarcinoma (239) or squamous cell carcinoma (240).

### 4.2 How Glutaminolysis in Cancer Cells Affect T-Cells

Engagement of the T-cell receptor and the co-stimulatory molecule CD28 triggers pathways under the control of transcription factors HIF-1α and mTOR, which not only increase glycolysis, but also upregulate the expression of amino acid transporters (7). Thus activated and proliferating T-cells also display increased glycolysis and glutaminolysis (94, 100, 241), which associates with the increased expression of SCL1A5 for glutamine (242), similar to cancer cells. *In vitro* experiments demonstrated that glutamine deprivation indeed reduces T-cell proliferation, suppresses differentiation towards Th1 phenotypes but stimulates regulatory FoxP3+ phenotypes (243). In addition to its role as intracellular antioxidant similar to cancer cells, glutathione in T-cells also supports mTOR and NFAT activation, thus driving glycolysis and glutaminolysis (92) and promoting inflammatory responses.

Blocking glutaminolysis in lung cancer cell lines results in upregulation of PD-L1 expression *via* NF-κB activity and dampened T-cell activation, but when glutaminolysis is inhibited together with PD-L1 blockade, the balance tips towards T-cell mediated cancer cell death (37).

Besides glucose, and glutamine, T cells also consume tryptophan. Deprivation of tryptophan can impair the function of these T cells (244). Pathologic conditions as hypoxia induce the presence of IDO on tumors, resulting in a significantly increased tryptophan metabolism by the kynurenine pathway (245). This increase of the metabolic product kynurenine is toxic for T-cells and leads to immunosuppression (246). Tryptophan 2,3-dioxygenase (TDO) is like IDO as it also catalyzes tryptophan into kynurenine (247, 248). Since IDO and TDO both convert tryptophan into kynurenine, they are both important targets to image this tryptophan metabolism.

### 4.3 Imaging Targets Related to Glutamine Metabolism

#### 4.3.1 Glutamine Metabolism

Glutamine metabolism in the TME can be visualized using glutamine radiolabelled with ^18^F or ^11^C (249–251). In preclinical experiments that studied the interplay between glutaminolysis and glycolysis, by using specific inhibitors in squamous cell lung cancer mouse models, PET imaging using [^18^F]FDG or [^11^C]Gln was used to quantify tumor metabolic profiles (240). In a lung cancer xenograft model, as well as in genetically engineered EGFR-mutant lung cancer model, increased [^18^F]Gln correlated with expression levels of SLC1A5 (252). Besides the fluorinated glutamine analogue, another PET tracer has been developed and tested *in vitro* and in animal models, namely L-[5-^11^C]-glutamine ([^11^C]Gln) (250). In contrast to [^18^F]Gln, this tracer is subjected to glutamase activity, converted to glutamic acid and further metabolized.

**Figure 5:**
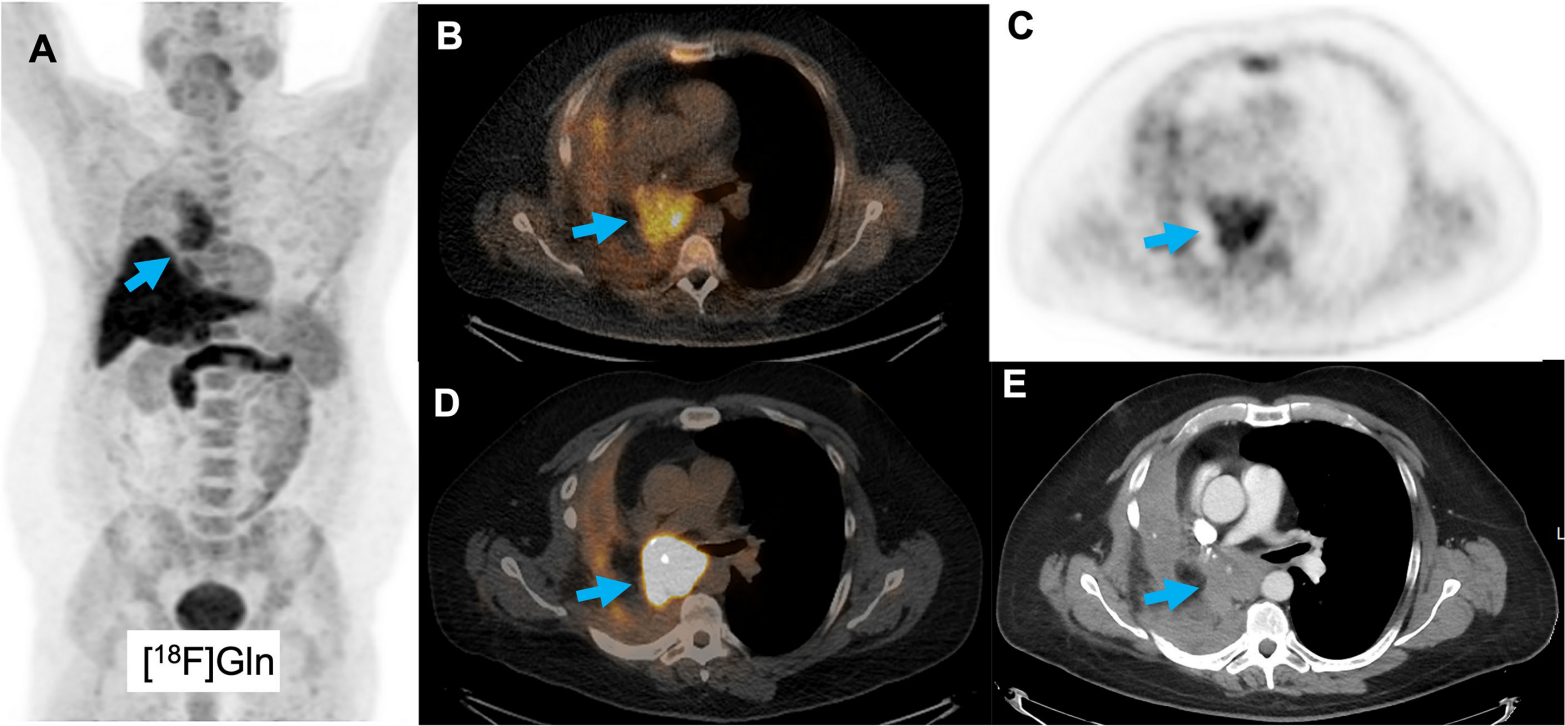
A patient with a squamous cell lung cancer lesion (arrow) scanned with [^18^F]Gln, showing increased uptake **(A–C)**. Corresponding [^18^F]FDG images show increased uptake as well **(D, E)**.

A clinical study in different cancer types, including lung cancer, supports the preclinical data that [^18^F]Gln (**Figure 5**) can be used as a biomarker of glutamine flux and metabolism in the TME (253–255). However, these studies focus on tumor detection and at present no clinical studies have incorporated glutamine-tracers to classify TME or monitor responses to immunotherapy. One clinical study showed a decrease in [^18^F]Gln uptake in the bone marrow upon chemotherapy with doxorubicin/rituximab, associated with a decrease in number of leukocytes (256). No clinical imaging studies are performed so far with [^11^C]Gln.

**Figure 6:**
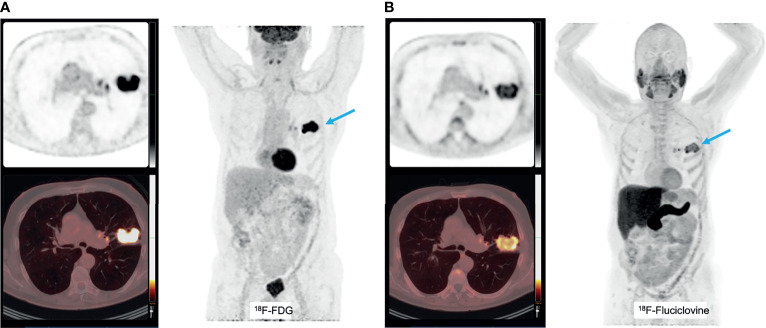
A patient with a T2bN0 primary adenocarcinoma of the left upper lobe, accidently detected on a [^18^F]Fluciclovine PET/CT scan for prostate cancer staging **(A)**. This tumor was PD-L1 negative and molecular analyses detected mutations in KEAP1, amplification in HER2 and CDK12 and no micro-satellite instability. The corresponding [^18^F]FDG PET images show increased uptake as well **(B)**.

Another tracer that can be a potential marker of glutamine metabolism is [^18^F]Fluciclovine, which is predominantly transported by the glutamine transporter SLC1A5. It is approved by the FDA as radiotracer for prostate malignancies (257), but its uptake is also increased e.g. breast cancer (258, 259) and it has preliminary been investigated to discriminate inflammatory lung lesions from lung cancer (260), with limited success. However, increased [^18^F]Fluciclovine uptake is anecdotally reported in squamous cell carcinoma and adenocarcinoma lung cancer (261), and complementary to [^18^F]FDG PET (**Figure 6**). Also, for this tracer, no studies in the context of immune therapy have yet been performed.

#### 4.3.2 Tryptophan Metabolism

Imaging of tryptophan metabolism and presence of IDO in the tumor metabolism is performed by using the clinical available α-[^11^C]methyl-L-tryptophan (AMT) PET tracer (262), in the context of breast, lung cancer and gliomas (263). One phase II study is enlisted on clinicaltrials.gov to investigate [^11^C]AMT as a predictive imaging biomarker of response to immunotherapy with the PD-1 inhibitor Pembrolizumab in melanoma patients (NCT03089606), but this study is not yet recruiting. As its short half-life of this tracer limits clinical application, other tryptophan analogues were developed and tested pre-clinically, such as 1-L-[^18^F]FETrp (264, 265), which will likely be translated to clinical setting.

## 5 Discussion

The incremental use of advanced technologies, such as metabolomics (51) or optical imaging (68), that yield in-depth information on a cellular level, has deepened our understanding of the complexity of tumor metabolism and its impact on other components of the tumor microenvironment. Metabolic adaptation is now an established hallmark of cancer (1) and NSCLC is no exception to this. Prevailing metabolic pathways in lung cancer, its’ counterpart in tumor infiltrating T-cells and its’ underlying regulatory mechanisms, including the role of immune checkpoint molecules, have been described in this review. Apart from advancing our insights in metabolic pathways in lung cancer cells and T-cells, these high-throughput cell-based technologies applied in *in vitro* studies implicitly pointed towards a role for *in vivo* molecular imaging in translating mechanistic insights into clinical applications. These *in vitro* studies illustrate that the complex interplay between cancer cells and immune cells cannot be fully recapitulated by cell cultures alone, as different metabolic processes might occur in the multi-cellular TME (266), as opposed to mono-cellular cultures. Studies in lung cancer have for example demonstrated that the source of carbon used to fuel mitochondrial metabolism is context dependent. *In vitro*, glutamine is the predominant carbon source for mitochondrial metabolism, whereas *in vivo*, glucose carbon contributes to a greater degree (22, 103, 239, 267). Furthermore, immune cells can to a certain extent adapt their metabolic pathways to tissue specific preferences (74), which is highly relevant when designing novel metabolic interventions to manipulate the TME to enhance anti-cancer immunity. Lastly, intra-tumoral co-existence of clones with differential metabolic dependencies is frequently observed in preclinical models, and mostly relates to impaired treatment outcomes (73). Both these assets, tissue specific immune metabolism and intra-tumoral heterogeneity, can best be investigated with the use of *in vivo* imaging.

As molecular imaging using PET has the potential to complement the current body of knowledge with information on *in vivo* processes in live subjects, tissue specific characteristics and the impact of regional differences in tumor metabolism, radiolabeled metabolic substrates are attractive tracers in the setting of a clinical study. These small molecules have the capacity to rapidly diffuse into tissues, accumulate intracellularly in target cells, often in direct relation to transporter expression and enzyme activity allowing easy kinetic modelling, and rapid clearance. These features translate to simple radiochemistry with short-lived tracers, such as ^18^F, with favorable target-to-background ratios obtained within short time frames of minutes to an hour and thus low effective dose for subjects. However, the complicating disadvantage of *in vivo* PET imaging of metabolic pathways in lung cancer is that in fact it quantifies the net result of the targeted metabolic pathway at rather low spatial resolution (268). It does not allow thorough assessment of relative contributions of cancer cells, supportive or immune cells.

**Figure 7:**
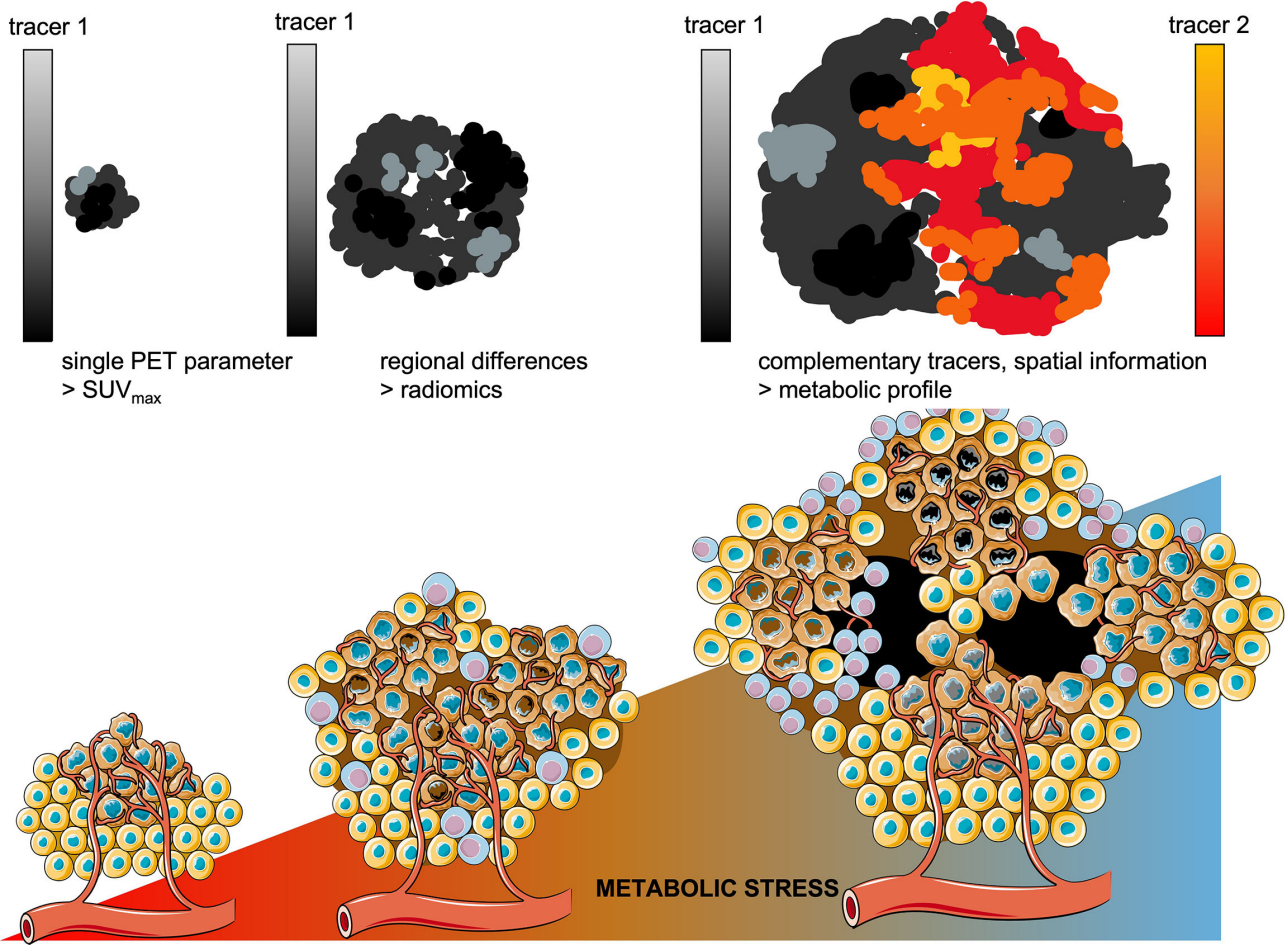
Towards metabolic profiling of lung cancer using PET/CT imaging. As cancer lesions progress, the metabolic stress increases which enforces metabolic competition between cancer cells and T-cells and drives further diversification of intra-tumoral regional differences. Whereas in smaller lesions single PET parameters might be sufficient to differentiate benign from malignant lesions. As the tumor lesions grow, radiomics are currently applied to quantitate the increasing intra-tumoral heterogeneity. Using complementary tracers, serial PET imaging would allow to address regional differences in dominant metabolic pathways, with conserved spatial information.

Despite this limitation, we envision a clear role for *in vivo* molecular imaging to advance the development of effective treatment for lung cancer in two domains. First, an imaging-based metabolic profile of a lung cancer lesion with conserved spatial information can optimize the efficacy of current standard of care treatments. The rapid clearance and short half-lives of tracers currently in use allow to perform consecutive PET scans with different tracers and thus providing a spatial profile of its dominant metabolic pathways (**Figure 7**). Such multi-modal imaging approach requires solid image registration techniques (269) as well as methods to quantify the correlative data. Although these required imaging processing techniques are yet under development, in principle such technology can be standardized and broadly implemented within current image processing platforms.

Instead of providing merely a summation of glucose metabolism in a lung cancer lesion, measured by its’ maximum uptake value (SUV_max_), [^18^F]FDG should be complemented with e.g.[^18^F]Gln or [^18^F]FMISO. When overlaying these quantitative PET-derived datasets, these measures of the downstream net results of regional metabolic interactions in lung cancer provide an impression whether the metabolic balance is tipped towards glucose dominant metabolism (tracer 1, e.g. [^18^F]FDG) or alternative/compensating metabolic pathways are active (tracer 2, e.g. [^18^F]Gln).

Current approach to assess intra-tumoral (metabolic) heterogeneity is *via* radiomics, which provides quantitative features that describe the distribution of signal intensities in a particular volume-of-interest. Indeed, increased intra-tumoral heterogeneity is inevitably linked to reduced overall survival (73, 270, 271) and this heterogeneity can be traced back to single cell level (272), providing a solid conceptual base for radiomics in lung cancer. Radiomics studies on intra-tumoral (contrast-enhanced) differences in tissue density on CT or differences in glucose metabolism on [^18^F]FDG PET, often identify a correlation between radiomics features and mutation status in lung cancer. Most studies have been insufficient to provide solid prediction upfront of responses to treatment (273–275). This can be explained by the complex interactions of regionally located sub-clones of lung cancer with other cellular components in the TME, as described in this review, which lack a direct link with radiomics-based measures of heterogeneity. Moreover, radiomics analyses lose spatial information, which is necessary to guide local treatments. Thus, imaging-based metabolic profiling of lung cancer based, with conserved spatial information of regional differences within a lesion, will therefore be complementary to radiomics and allow tailoring treatment on a regional level in the tumor (276).

For example, in individualized radiotherapy planning, such information would enable radiotherapy planning based on intra-tumoral regional differences and adapt the radiation portal prior to treatment (277) or during treatment (278). Since ‘dose-painting’ is increasingly applied in lung cancer, especially in early-stage lung cancer (62, 279) local ablative (stereo-tactic) radiotherapy is considered a reasonable alternative to surgery. The imaging-based metabolic profile of an individual lung tumor may allow personalized dose prescription resulting in minimal toxicity with maximal chance of control in lung cancer. For the locally advanced stage, comprehensive metabolic profiling of lung cancer using a dual-tracer approach might allow selection of patients who will benefit from metabolic interventions accompanying chemoradiotherapy. Previous studies failed to demonstrate benefit in a randomized, unselected approach (280), and the existence of metabolic heterogeneity in a lung cancer lesion is deemed one of the underlying reasons underscoring the necessity to select patients based on the intervention that is addressed.

Second, the metabolic TME is one of the major determinants of an immune suppressive microenvironment for tumor-infiltrating T-cells, and T-cell metabolism is regulated by druggable immune checkpoint molecules such as PD-1. Therefore, complementary to immune imaging, imaging-based metabolic profiling also holds potential in metastatic setting. During PD-1/PD-L1 targeting therapy, tumor-infiltrating T-cells find themselves entangled between the metabolic constraints of the TME and the unleashed potential to accelerate cellular metabolism and execute their cytotoxic function. The incomplete understanding of which metabolic pathways are actual in a particular patient with lung cancer and its’ intra-tumoral regional differences, is likely one of the reasons why current response rates are usually below 50%, and for most patients, long-term survival is not the reality (281). For example, if hypoxia is dominating the metabolic TME, adding CD28 blockade by anti-CTLA4 monoclonal antibody to anti- PD-1 therapy might yield higher clinical benefit than in patients where hypoxia is relatively less, and PD-1 inhibition is sufficient to reinvigorate T-cells. In the first line setting, monotherapy immunotherapy, chemo-immunotherapy with and without angiogenesis inhibition (282), chemo-immunotherapy (283) as well as immunotherapy doublets have become available (284). Except for PD-L1 status, current selection for a certain treatment regimen is usually based on national/local standards and preferences. We postulate that imaging-based metabolic profiling can provide an additional role to rationally choose first-line treatment, increase its’ efficacy and avoid unnecessary exposure to potential adverse effects.

In addition, the trial-and-error approach in developing novel (combination) immunotherapies is failing (285) and new tools for smarter drug-development pipelines are mandatory. Upon progression on first-line therapy, multiple studies with new immunomodulatory compounds are ongoing, including metabolic interventions (56), usually in a “one-size-fits-all” approach. Complementary to platform trials (e.g. HUDSON (NCT03334617)), attrition rates can probably be improved the metabolic TME is taken into consideration, and results from the few studies that incorporated molecular imaging of metabolic pathways are eagerly awaited.

In conclusion, to advance the treatment landscape of lung cancer, molecular imaging of the metabolic TME should be integrated, as a biomarker tool to support the rational select current treatments and design of next generation of clinical trials.

## Author’s Note

The figures are originally created with use of open-source templates from Servier Medical Art under Creative Commons Attribution 3.0 Unported License, *via* smart.servier.com

## Author Contributions

LH and EA drafted the outline of the review. EG and JW performed the literature search and reviewed the retrieved articles. All authors participated in analyzing and writing. All authors contributed to the article and approved the submitted version.

## Funding

SH received Dutch Cancer Society grant KWF-YIG10099. EA received Dutch Cancer Society grant KWFYIG12493. MK received funding from Cluster of Excellence iFIT (EXC 2180) “Image Guided and Functionally Instructed Tumor Therapies”, University of Tuebingen, Tübingen, Germany.

## Conflict of Interest

MH received research grants from AstraZeneca, BristolMeyerSquibb, Janssen Pharmaceutica, Stichting Treatmeds, Merck, MSD, Novartis, Pamgene, Pfizer, Roche, Roche diagnostics, and received fees from Abbvie, Astrazeneca, BMS, Lilly,MSD, Novartis, Pfizer, Roche, none related to this manuscript.

The remaining authors declare that the research was conducted in the absence of any commercial or financial relationships that could be construed as a potential conflict of interest.

## Publisher’s Note

All claims expressed in this article are solely those of the authors and do not necessarily represent those of their affiliated organizations, or those of the publisher, the editors and the reviewers. Any product that may be evaluated in this article, or claim that may be made by its manufacturer, is not guaranteed or endorsed by the publisher.
